# Effects of long-term inhaled corticosteroids on growth velocity and adult height in children with asthma: a systematic review and meta-analysis

**DOI:** 10.3389/fmed.2026.1937498

**Published:** 2026-09-14

**Authors:** Jinguang Zhao, Aiai Dong, Yibu Kong, Tianlong Xie, Xinyan Yu, Baoxin Li, Tianbao Tan, Jiqiang Mi, Yinan Guo

**Affiliations:** 1 College of Traditional Chinese Medicine, Changchun University of Chinese Medicine, Changchun, Jilin, China; 2 Department of Pediatrics, The Affiliated Hospital to Changchun University of Chinese Medicine, Changchun, Jilin, China; 3 Post-Doctoral Research Center, Jilin Aodong Pharmaceutial Group Company Limited, Dunhua, Jilin, China

**Keywords:** adult height, asthma, children, growth velocity, inhaled corticosteroids, meta-analysis, systematic review

## Abstract

**Background:**

Inhaled corticosteroids (ICS) are the cornerstone of long-term controller therapy for childhood asthma, but their effects on growth remain controversial.

**Objective:**

To evaluate the effects of long-term maintenance ICS therapy on linear growth velocity and adult or near-adult height in children with asthma.

**Methods:**

We conducted a systematic review and meta-analysis in accordance with PRISMA 2020. The review was registered in PROSPERO on 27 March 2026 (CRD420261351837), after completion of the database searches on 26 March 2026 but before final study selection, data extraction, risk-of-bias assessment, and quantitative synthesis. PubMed/MEDLINE, Embase, CENTRAL, CNKI, WanFang, and SinoMed were searched from inception to March 26, 2026, with supplementary searches of ClinicalTrials.gov, WHO ICTRP, and reference lists. Eligible studies included children and adolescents with asthma receiving ICS for at least 6 months. Random-effects models were used to pool mean differences (MDs) with 95% confidence intervals (CIs).

**Results:**

Thirteen studies were included, and six study reports contributed to at least one quantitative analysis. In the primary random-effects meta-analysis, ICS treatment was associated with a non-significant reduction in linear growth velocity compared with controls (MD, −0.38 cm/year; 95% CI, −0.84 to 0.07; I^2^ = 64%). After excluding studies judged to be at high risk of bias, the pooled estimate became statistically significant (MD, −0.59 cm/year; 95% CI, −1.10 to −0.07), suggesting a possible small reduction in linear growth velocity rather than definitive evidence of an effect. A reduction was observed in school-age children (MD, −0.50 cm/year; 95% CI, −0.96 to −0.04), although age-specific differences remained uncertain. Limited long-term evidence suggested a small reduction in adult height (MD, −1.20 cm; 95% CI, −1.90 to −0.50).

**Conclusion:**

In the primary analysis, long-term maintenance ICS therapy did not demonstrate a statistically significant reduction in linear growth velocity in children with asthma. However, sensitivity analysis excluding studies at high risk of bias suggested a possible small reduction. Evidence regarding adult height was limited and derived predominantly from one long-term follow-up study. These findings should therefore be interpreted cautiously and balanced against the established benefits of ICS for asthma control.

## 1 Introduction

Childhood asthma is a common chronic disease, and inhaled corticosteroids (ICS) are the recommended first-line controller therapy for persistent disease. However, concern about possible growth suppression remains a major barrier to long-term adherence (1–4).

Previous studies and follow-up reports have reported inconsistent findings: some showed reduced linear growth velocity, especially early in treatment, whereas others suggested limited effects on final height or partial catch-up growth (5–10). Several high-quality systematic reviews and meta-analyses, including Loke et al. (11) and Cochrane reviews (12, 13), have provided important evidence regarding the effects of ICS on linear growth, dose-response relationships, and potential differences among ICS drugs and delivery devices. However, important knowledge gaps remain. First, much of the existing evidence has focused primarily on linear growth velocity during treatment, whereas evidence on adult or near-adult height remains limited and requires careful synthesis with short-term growth outcomes. Second, clinical and methodological heterogeneity related to age, ICS formulation, dose, treatment duration, comparator type, and outcome definitions continues to complicate interpretation. Third, updated searches incorporating both international and Chinese databases, trial registries, risk-of-bias assessment, and certainty-of-evidence evaluation may help provide a more current and transparent appraisal of the available evidence.

Therefore, building on previous systematic reviews and Cochrane analyses, we conducted an updated systematic review and meta-analysis to evaluate the effects of long-term maintenance ICS therapy on both linear growth velocity and adult or near-adult height in children with asthma. Compared with previous reviews, the present study aimed to provide an updated and focused synthesis of randomized trials and long-term follow-up reports, incorporate searches of both international and Chinese databases, assess risk of bias using RoB 2, evaluate certainty of evidence using GRADE, and explore potential sources of heterogeneity, including age group, ICS type, dose, treatment duration, comparator type, and overall risk of bias (11–15).

## 2 Methods

### 2.1 Study design and registration

This systematic review and meta-analysis followed the Cochrane Handbook and PRISMA 2020 statement. The review was registered in PROSPERO on 27 March 2026 (CRD420261351837), after completion of the database searches on 26 March 2026 but before final study selection, data extraction, risk-of-bias assessment, and quantitative synthesis. Therefore, the registration was retrospective with respect to the literature search but was completed before subsequent review procedures and analyses (16).

### 2.2 Inclusion and exclusion criteria

Eligibility criteria were defined according to the PICOS framework. We included randomized controlled trials and long-term follow-up reports derived from original randomized trial cohorts involving children or adolescents aged <18 years with asthma (17). Asthma had to be diagnosed according to recognized clinical or guideline-based criteria, and participants were required to be clinically stable at baseline (18). The intervention of interest was long-term maintenance inhaled corticosteroid (ICS) therapy for at least 6 months, regardless of formulation, dose, or inhalation device. The threshold of at least 6 months was selected to capture maintenance rather than short-term, rescue-only, or intermittent ICS exposure and to allow sufficient follow-up for measurable changes in linear growth velocity. This threshold was not intended to define a specific dose category; therefore, studies were eligible regardless of whether the ICS regimen was low-, medium-, or high-dose, provided that the treatment represented regular maintenance therapy. Studies evaluating only short-course, rescue-only, or intermittent ICS use without a sustained maintenance component were not considered eligible for the main synthesis. Eligible comparators included placebo, nonsteroidal controller medications such as leukotriene receptor antagonists, or no regular long-term controller therapy (19). The primary outcomes were linear growth velocity and adult or near-adult height; secondary outcomes included height SDS/Z-score, body weight, body mass index, and reported growth-related adverse events.

We restricted the main inclusion criteria to randomized trials and long-term follow-up reports derived from randomized trial cohorts because the objective of this review was to estimate the comparative effect of maintenance ICS therapy on growth-related outcomes while minimizing confounding by indication and disease severity. Although prospective cohort studies can provide valuable long-term safety information, growth outcomes in observational studies may be influenced by asthma severity, disease control, systemic inflammation, pubertal timing, nutritional status, adherence, inhalation technique, and cumulative exposure to oral corticosteroids. Therefore, independent non-randomized cohort studies were not included in the main synthesis to avoid combining evidence with substantially different risks of confounding and methodological heterogeneity.

Articles published in English or Chinese were eligible. We excluded studies involving healthy controls, cross-sectional studies, retrospective studies, prospective cohort studies unrelated to original randomized trial cohorts, case reports, case series, reviews, editorials, conference abstracts, and animal studies. We also excluded studies in which other conditions or medications could substantially confound growth outcomes.

### 2.3 Literature search strategy

We searched PubMed/MEDLINE, Embase, CENTRAL, Scopus, CNKI, WanFang, SinoMed, ClinicalTrials.gov, and WHO ICTRP from inception to March 26, 2026, and also screened reference lists. The database searches were completed on 26 March 2026, 1 day before PROSPERO registration on 27 March 2026. Search terms covered asthma, children, inhaled corticosteroids, and growth. Full search strategies, including the additional Scopus search, are provided in Supplementary Appendix 1.

### 2.4 Study selection and data extraction

#### 2.4.1 Study selection

All records identified through the searches were first imported into EndNote software for deduplication. Two reviewers then independently screened titles and abstracts and subsequently conducted full-text review of potentially eligible studies. Disagreements were resolved through discussion; when consensus could not be reached, a third reviewer made the final decision. The study selection process was presented in a PRISMA flow diagram.

#### 2.4.2 Data extraction

Two reviewers independently extracted data using a standardized form and cross-checked the results. Extracted information included study characteristics, participant characteristics, intervention and comparator details, growth-related outcomes, and information required for risk-of-bias assessment. For outcome data reported only in graphical form, WebPlot Digitizer was used where necessary. No study authors were contacted because the information required for eligibility assessment and quantitative synthesis was available from the published reports. No unpublished data were requested or incorporated into this review. When multiple reports were derived from the same trial cohort, we treated them as linked reports rather than independent studies. Each trial cohort contributed only once to any single meta-analysis. Original trial reports were used for short- or medium-term outcomes such as linear growth velocity, whereas long-term follow-up reports were used only when they provided distinct outcomes, such as adult or near-adult height. This approach was used to avoid double counting of participants within the same outcome analysis.

### 2.5 Risk-of-bias assessment

Risk of bias was independently assessed by two reviewers, and disagreements were resolved through discussion or consultation with a third reviewer when necessary. For randomized controlled trials, we used the revised Cochrane risk-of-bias tool for randomized trials (RoB 2). Because RoB 2 is outcome-specific, risk of bias was assessed with linear growth velocity/height-related outcomes as the outcome of interest.

The following five domains were evaluated: (1) bias arising from the randomization process; (2) bias due to deviations from intended interventions; (3) bias due to missing outcome data; (4) bias in measurement of the outcome; and (5) bias in selection of the reported result. Each domain was judged as “low risk of bias,” “some concerns,” or “high risk of bias,” and an overall risk-of-bias judgment was assigned for each study according to the RoB 2 algorithm. For long-term follow-up reports derived from original randomized trials, risk of bias was assessed according to the extent to which the reported growth outcome still reflected the original randomized comparison.

### 2.6 Data synthesis and statistical analysis

Mean difference (MD) with 95% confidence interval (CI) was used for outcomes measured on comparable scales; standardized mean difference (SMD) was used otherwise. Random-effects models were applied because of anticipated clinical and methodological heterogeneity. Heterogeneity was assessed with Cochran’s Q test and the I^2^ statistic. Prespecified subgroup analyses were planned by age group, ICS type, dose, treatment duration, comparator type, study design, and overall risk of bias. Because of the limited number of studies and incomplete reporting of cumulative dose, adherence, and treatment duration, dose-response analyses and meta-regression could not be reliably performed. Sensitivity analyses excluded studies at high risk of bias, omitted studies sequentially using a leave-one-out approach, and compared analytical models. Publication bias was assessed only when at least 10 studies were available. Certainty of evidence for primary outcomes was rated using GRADE. EndNote 2025 was used for literature management, RevMan 5.4 for quantitative synthesis, and RStudio 4.4.3 with robvis for risk-of-bias visualization.

## 3 Results

### 3.1 Results of literature screening

A total of 1,719 potentially relevant records were identified through systematic searches of PubMed, Embase, the Cochrane Library, Scopus, CNKI, WanFang Data, and SinoMed. No additional eligible studies were identified through manual reference screening, trial registry searches, Scopus screening, or other sources. After removal of 801 duplicate records, 918 records remained for title and abstract screening. Following title and abstract screening, 27 articles were assessed in full text. Fourteen studies were excluded after full-text review for not meeting the eligibility criteria, and 13 studies were ultimately included in the systematic review. Of these, six study reports contributed data to at least one quantitative analysis, whereas the remaining seven studies were retained in the qualitative synthesis only because of differences in outcome definitions, incomplete reporting of summary statistics, or insufficient methodological comparability. The study selection process is shown in Figure 1.

**FIGURE 1:**
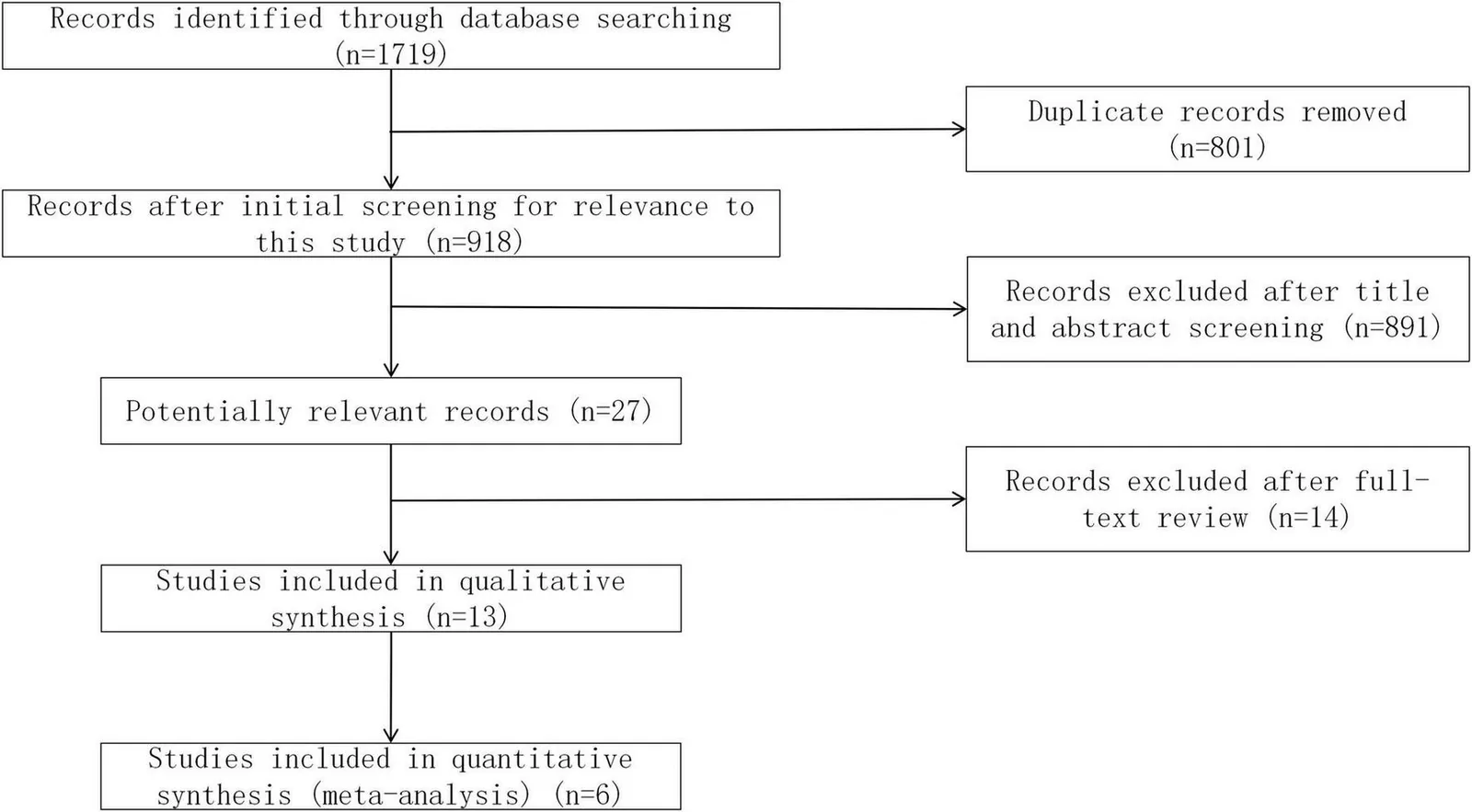
Flowchart of literature search and study selection.

### 3.2 Basic characteristics of the included studies

Details of the included studies are summarized in Table 1. A total of 13 studies met the eligibility criteria for this review (5, 8–10, 18, 20–27). The included studies were predominantly randomized controlled trials, including one long-term follow-up report derived from an original RCT (9). These studies were conducted in China, the United States, the Netherlands, the United Kingdom, and across multinational multicenter settings. The study populations covered a broad age range, including infants, preschool children, school-age children, and prepubertal children. The interventions mainly involved long-term maintenance ICS therapy, with commonly used agents including beclomethasone dipropionate, fluticasone propionate, and budesonide. Comparators included placebo, no ICS treatment, nonsteroidal controller therapies, and montelukast. Follow-up duration ranged from 6 months to 6 years. Growth-related outcomes varied across studies and included linear growth velocity, growth increment, height standard deviation score, height change during treatment, and adult height. Owing to substantial heterogeneity in outcome definitions, reporting formats, and completeness of statistical parameters, six study reports contributed to at least one quantitative analysis, whereas seven studies were retained in the qualitative synthesis only. Specifically, Allen, Doull, Piao, and Wang contributed data to the primary analysis of linear growth velocity; Becker contributed data to the comparison between ICS and montelukast for linear growth velocity; and Kelly contributed data to the adult height analysis. The remaining seven studies were retained in the qualitative synthesis only, and the reasons for non-inclusion in quantitative synthesis are provided in Supplementary Appendix 2.

**TABLE 1:** Main characteristics of the included studies.

Study	Design	Population	Sample size, I/C	ICS regimen	Comparator	Follow-up	Main growth outcome	Included in quantitative synthesis	Overall RoB 2 judgment
Chen et al., (20)	RCT	Children with mild asthma	10/10	Beclomethasone dipropionate 200/400 μg/day	Placebo	1 year	Height SDS and BMD	No	Some concerns
Piao, (21)	RCT	Infants with asthma	51/17	Beclomethasone dipropionate 50/100 μg/day	Non-ICS treatment	6–12 months	linear growth velocity	Yes	High risk
Wang et al., (22)	RCT	Children with asthma	29/14	Beclomethasone dipropionate 100–400 μg/day	No ICS	6–8 months	Growth velocity and BMD	Yes	High risk
Allen et al., (10)	RCT	Prepubertal children	219/106	Fluticasone propionate 50/100 μg twice daily	Placebo	1 year	Height and linear growth velocity	Yes	Some concerns
Becker et al., (23)	RCT	Prepubertal children	119/121	Beclomethasone dipropionate 200 μg twice daily	Placebo/Montelukast	56 weeks	linear growth velocity	Yes	Low risk
Doull et al., (5)	RCT	Children aged 7–9 years	50/44	Beclomethasone dipropionate 400 μg/day	Placebo	7 months	linear growth velocity	Yes	High risk
Guilbert et al., (18)	RCT	Preschool children	143/142	Fluticasone propionate 88 μg twice daily	Placebo	2 years	Height	No	Low risk
Kelly et al., (9)	Long-term follow-up of RCT	Children followed into adulthood	311/418	Budesonide 400 μg/day during childhood	Placebo	4–6 years plus long-term follow-up	Adult height	Yes	High risk
Martinez et al., (24)	RCT	Children/adolescents	143/74	Daily or rescue beclomethasone dipropionate	Placebo	44 weeks	Height	No	Some concerns
Sorkness et al., (25)	RCT	Children with asthma	190/95	Fluticasone propionate ± LABA	Montelukast	48 weeks	Growth	No	Some concerns
CAMP, (8)	RCT	Children with asthma	311/418	Budesonide 400 μg/day	Placebo	4–6 years	Height	No	Low risk
Visser et al., (26)	RCT	Children with asthma	27/28	Fluticasone propionate, step-down regimen	Fixed-dose fluticasone propionate	1 year	Height	No	Some concerns
Visser et al., (27)	RCT	Children with asthma	27/28	Fluticasone propionate, high-to-low step-down regimen	Fixed-dose fluticasone propionate	2 years	linear growth velocity	No	Some concerns

BDP, beclomethasone dipropionate; BMD, bone mineral density; FP, fluticasone propionate; ICS, inhaled corticosteroids; LABA, long-acting beta2-agonist; SDS, standard deviation score.

### 3.3 Risk-of-bias assessment

Using the RoB 2 tool and assessing bias with linear growth/height-related outcomes as the outcome of interest, the overall risk of bias of the included studies ranged from low risk to high risk. In general, the more recent multicenter double-blind randomized trials were judged to be at low risk of bias or to raise some concerns, whereas several earlier studies were judged to have high risk of bias.

The main concerns arose from insufficient detail on the randomization process, lack of blinding or inadequate control of deviations from intended interventions, missing outcome data or post-randomization exclusions, and limited information on prespecification of reported growth outcomes. By contrast, most studies were judged to have relatively low risk of bias in measurement of the outcome, because height and linear growth velocity were generally assessed using standardized objective methods. The detailed study-level and domain-level RoB 2 judgments are presented in Figures 2A, B.

**FIGURE 2:**
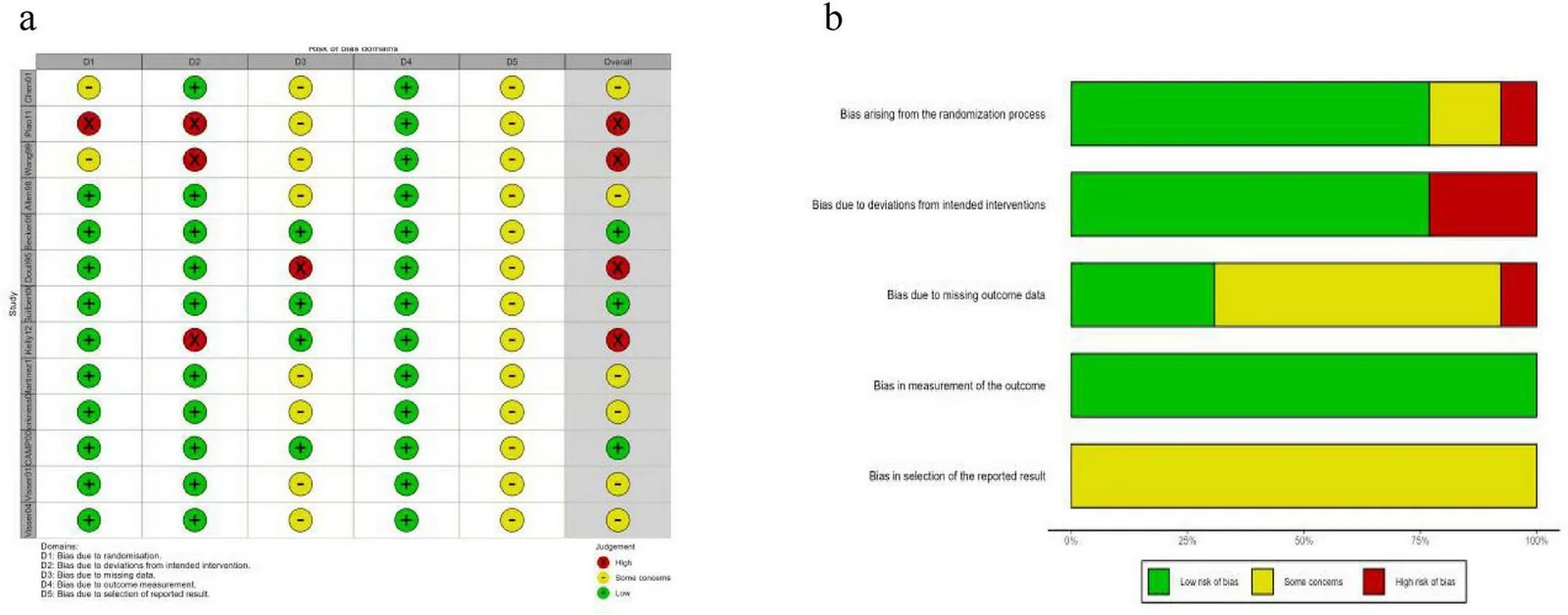
Risk of bias summary and proportions across domains. **(a)** Risk-of-bias judgments for each included randomized controlled trial using RoB 2 for height-related outcomes. Review authors’ judgments for each RoB 2 domain in each included randomized controlled trial are shown. Green indicates low risk of bias, yellow indicates some concerns, and red indicates high risk of bias. **(b)** Distribution of RoB 2 judgments across domains for height-related outcomes among the included randomized controlled trials. The percentages of studies judged as having low risk of bias, some concerns, or high risk of bias in each RoB 2 domain are shown. Green indicates low risk of bias, yellow indicates some concerns, and red indicates high risk of bias.

### 3.4 Primary outcome: Linear growth velocity

Six comparisons from four study reports contributed data to the primary quantitative synthesis of linear growth velocity. Meta-analysis using a random-effects model showed that, compared with controls, ICS treatment was associated with a non-significant reduction in linear growth velocity (MD = −0.38 cm/year, 95% CI −0.84 to 0.07; *P* = 0.10; I^2^ = 64%). Moderate heterogeneity was observed across studies. The forest plot is shown in Figure 3A.

**FIGURE 3:**
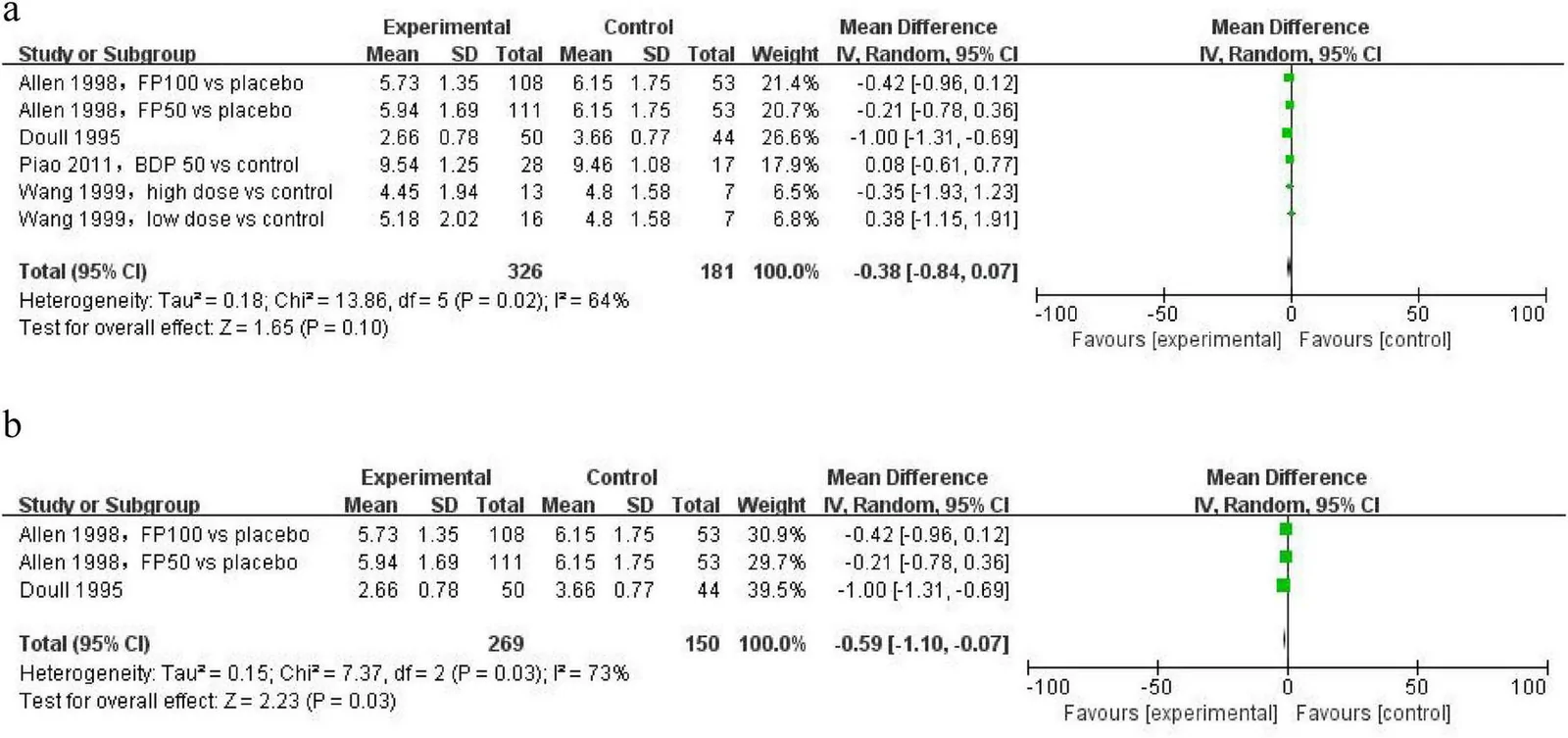
Forest plots of primary analysis and sensitivity analysis. **(a)** Forest plot for the meta-analysis of the effect of inhaled corticosteroids on linear growth velocity in children. The pooled effect size is presented as mean differences (MDs) with 95% confidence intervals (CIs) using a random-effects model. Negative values indicate lower linear growth velocity in the ICS group than in the control group. **(b)** Forest plot for the sensitivity analysis after exclusion of studies with high risk of bias. The robustness of the pooled estimate was assessed by excluding studies with high risk of bias. Effect sizes are presented as mean differences (MDs) with 95% confidence intervals (CIs) using a random-effects model. Negative values indicate reduced linear growth velocity in the inhaled corticosteroid group compared with the control group.

### 3.5 Sensitivity analysis

Sensitivity analysis was performed by excluding studies judged to be at high risk of bias. After exclusion of these studies, the pooled difference in linear growth velocity between the ICS and control groups became statistically significant (MD = −0.59 cm/year, 95% CI −1.10 to −0.07; *P* = 0.03). Sequential leave-one-out analyses of the primary outcome showed pooled MDs ranging from −0.50 to −0.20 cm/year. The direction of effect remained toward lower growth velocity in the ICS group in all leave-one-out analyses, but statistical significance was not consistent: the 95% CI excluded the null only when Piao et al. was omitted (MD = −0.50 cm/year, 95% CI −0.96 to −0.04), whereas the remaining leave-one-out estimates crossed the null. The 95% prediction interval for the primary random-effects analysis was −1.71 to 0.95 cm/year, indicating that the effect expected in a future comparable study could range from a reduction in linear growth velocity to little or no difference. Because statistical significance was observed only in selected sensitivity analyses and the prediction interval crossed the line of no effect, these results should be interpreted as supportive evidence suggesting a possible small effect rather than definitive evidence that ICS reduces linear growth velocity. The sensitivity analysis results are shown in Figure 3B, and full leave-one-out results are provided in Supplementary Appendix 3.

### 3.6 Subgroup analysis

#### 3.6.1 By age group

In the age-based subgroup analysis, ICS treatment was associated with reduced linear growth velocity within the school-age subgroup (MD = −0.50 cm/year, 95% CI −0.96 to −0.04; *P* = 0.03), whereas no significant difference was observed within the infant subgroup (MD = 0.08 cm/year, 95% CI −0.61 to 0.77; *P* = 0.82). However, the formal test for subgroup differences was not statistically significant (*P* = 0.17). Therefore, although a significant effect was observed within school-age children, the available evidence is insufficient to conclude that age modifies the treatment effect or that school-age children are more susceptible than infants. The results are shown in Figure 4A.

**FIGURE 4:**
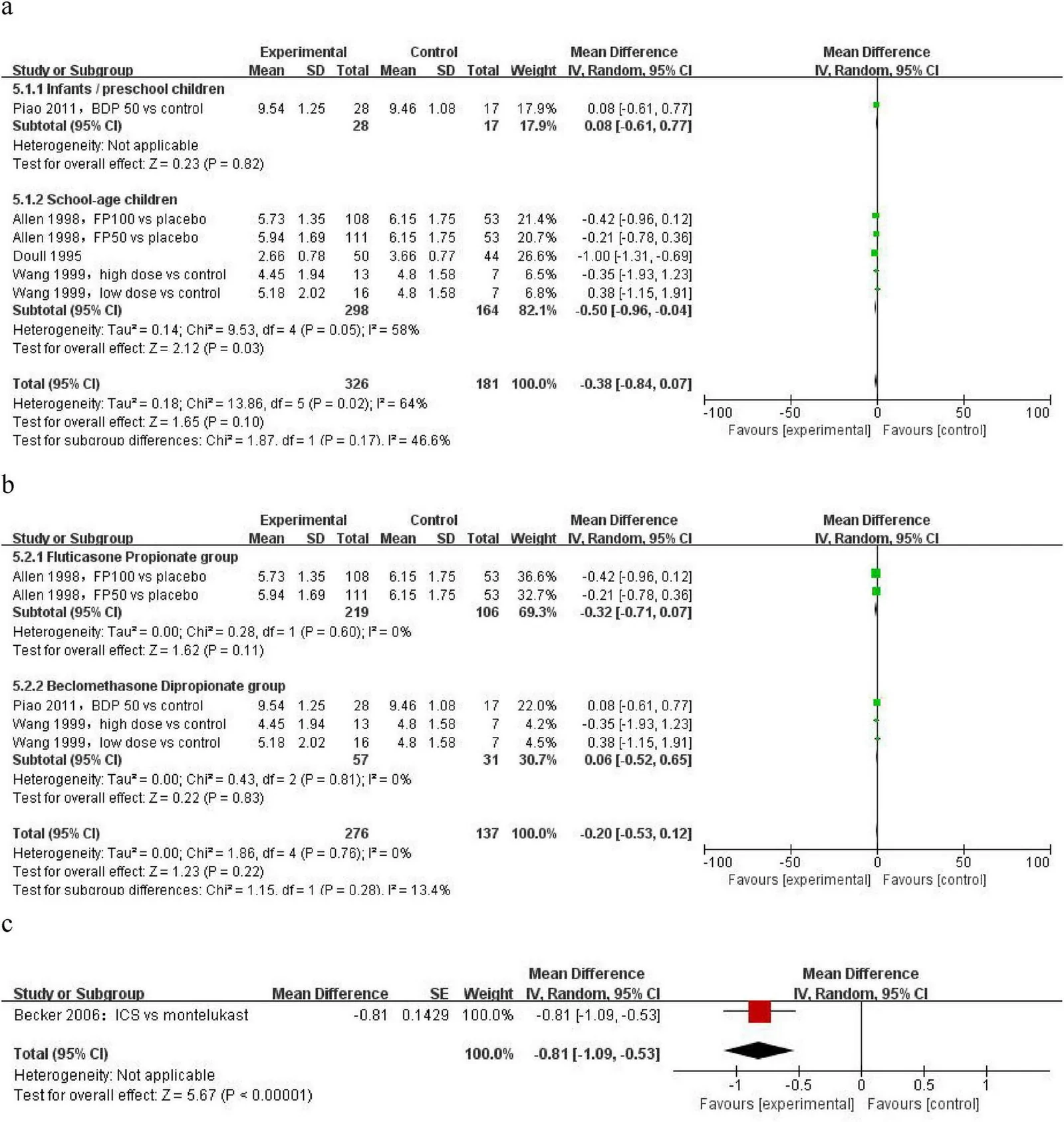
Subgroup and comparator analyses for linear growth velocity. **(a)** Forest plot for the subgroup analysis of the effect of inhaled corticosteroids on linear growth velocity in different age groups. The effect of inhaled corticosteroids (ICS) on linear growth velocity was evaluated separately in different age subgroups. Effect sizes are presented as mean differences (MDs) with 95% confidence intervals (CIs) using a random-effects model. Negative values indicate reduced linear growth velocity in children receiving ICS compared with controls. **(b)** Forest plot of subgroup analyses by inhaled corticosteroid type. The effect of different inhaled corticosteroid (ICS) agents on linear growth velocity was evaluated in subgroup analyses. Pooled mean differences (MDs) with 95% confidence intervals (CIs) were estimated using a random-effects model. Negative values indicate reduced linear growth velocity in children treated with ICS compared with controls. **(c)** Forest plot for the comparison of inhaled corticosteroids versus montelukast on linear growth velocity in children. The effect size is presented as mean difference (MD) with 95% confidence interval (CI). Negative values indicate reduced linear growth velocity in children treated with inhaled corticosteroids compared with those treated with montelukast.

### 3.6.2 By drug type

In subgroup analyses by ICS type, the pooled effect estimate was MD = −0.32 cm/year (95% CI −0.71 to 0.07; *P* = 0.11) for fluticasone and MD = 0.06 cm/year (95% CI −0.52 to 0.65; *P* = 0.83) for beclomethasone, with no statistically significant difference between subgroups (*P* = 0.28). Because of the small number of studies and variation in dose, delivery device, adherence, and follow-up duration, the absence of statistically significant subgroup differences should not be interpreted as evidence of equivalent growth effects across ICS formulations. The results are shown in Figure 4B.

### 3.7 Comparison between ICS and montelukast

One randomized controlled trial compared ICS with montelukast in terms of linear growth velocity in children. In that study, linear growth velocity was lower in the ICS group than in the montelukast group (MD = −0.81 cm/year, 95% CI −1.09 to −0.53; *P* < 0.00001). Because this comparison was based on a single study, the finding should be interpreted cautiously. The results are shown in Figure 4C.

### 3.8 Adult height

Evidence on adult height was derived predominantly from the long-term follow-up report of the CAMP study (Kelly et al., 2012) (9). Compared with placebo, individuals who had received ICS treatment during childhood had a lower adult height (MD = −1.20 cm, 95% CI −1.90 to −0.50). Because this outcome was based largely on a single long-term follow-up cohort, the finding should be interpreted cautiously and should not be considered definitive evidence that ICS therapy reduces final adult height across all pediatric asthma populations.

Formal assessment of publication bias was not performed for the main outcomes because fewer than 10 studies were available for each pooled analysis. Therefore, funnel plots and Egger’s test were not considered informative in the present review.

### 3.9 Certainty of evidence

According to the GRADE assessment, the certainty of evidence for the overall effect of ICS on linear growth velocity was low, mainly because of risk of bias in some studies, inconsistency across studies, and imprecision related to limited sample size. The certainty of evidence for the comparison between ICS and montelukast was rated as moderate; however, because this estimate was derived from a single randomized controlled trial, it should still be interpreted cautiously. The certainty of evidence for adult height was also rated as moderate; however, because this estimate was derived predominantly from a single long-term follow-up study, it should likewise be interpreted with caution. The certainty of evidence for the age subgroup analysis ranged from low to very low. The GRADE results for the primary outcomes are presented in Table 2.

**TABLE 2:** GRADE certainty of evidence for the primary outcomes.

Outcome	Interpretation	Effect estimate	Study design	Risk of bias	Inconsistency	Indirectness	Imprecision	Publication bias	Certainty of evidence	Interpretation
Linear growth velocity: ICS vs. placebo/no regular controller	ICS may reduce linear growth velocity, but the evidence is not robust	MD −0.38 cm/year (95% CI −0.84 to 0.07)	RCTs	Serious	Serious	Not serious	Serious	Not assessable	Low	ICS may reduce linear growth velocity, but the evidence is not robust
Linear growth velocity: ICS vs. montelukast	Compared with montelukast, ICS may be associated with lower linear growth velocity	MD −0.81 cm/year (95% CI −1.09 to −0.53)	RCT	Not serious*	Not serious	Not serious	Not serious	Not assessable	Moderate	Compared with montelukast, ICS may be associated with lower linear growth velocity
Adult height: ICS vs. placebo	ICS may be associated with a small reduction in adult height	MD −1.20 cm (95% CI −1.90 to −0.50)	Long-term follow-up of RCT	Serious	Not serious	Not serious	Not serious	Not assessable	Moderate	ICS may be associated with a small reduction in adult height
Linear growth velocity in school-aged children (age subgroup)	Growth suppression may be more apparent in school-aged children	MD −0.50 cm/year (95% CI −0.96 to −0.04)	RCTs	Serious	Serious	Not serious	Serious	Not assessable	Low	Growth suppression may be more apparent in school-aged children

*Although the single RCT comparing ICS with montelukast showed a precise effect estimate, the certainty of evidence was conservatively rated as moderate because the result was based on a single study and publication bias could not be assessed.

## 4 Discussion

### 4.1 Main findings

In this systematic review and meta-analysis, the primary random-effects analysis did not demonstrate a statistically significant reduction in linear growth velocity among children receiving long-term maintenance ICS therapy. A statistically significant reduction was observed only in the sensitivity analysis after excluding studies judged to be at high risk of bias, indicating that the pooled estimate was sensitive to study selection and risk-of-bias considerations. Therefore, the current evidence suggests a possible small adverse effect of ICS on growth velocity, but it does not provide definitive or robust confirmation of such an effect.

A reduction was observed within the subgroup of school-age children; however, the formal test for subgroup differences was not statistically significant, and age-specific susceptibility should therefore be interpreted cautiously. Limited long-term evidence suggested a small reduction in adult height, but this finding was derived predominantly from a single long-term follow-up study. Overall, the available evidence indicates that any potential growth-related effect of long-term ICS therapy is likely to be small, uncertain, and dependent on study quality and design.

In relation to previous systematic reviews and Cochrane analyses, the present review should be interpreted as an updated and complementary synthesis rather than as a replacement of earlier work. Loke et al. and relevant Cochrane reviews established that ICS may affect growth in children with persistent asthma, including possible dose-response effects and differences among ICS molecules and delivery devices. Our review adds to this literature by focusing specifically on long-term maintenance ICS exposure, integrating evidence on both linear growth velocity and adult or near-adult height, applying RoB 2 and GRADE to reassess risk of bias and certainty of evidence, and distinguishing the primary analysis from sensitivity, subgroup, and long-term follow-up findings. This approach allows a more cautious clinical interpretation of the available evidence, particularly because the primary pooled analysis was not statistically significant and some conclusions depend on sensitivity analyses or limited long-term data.

### 4.2 Interpretation and mechanisms

A potential effect of ICS on growth is biologically plausible. Corticosteroids may influence height gain through effects on the growth hormone–insulin-like growth factor axis, bone metabolism, and growth plate function (3, 28). However, interpretation of the pooled estimates is complicated by substantial clinical heterogeneity across the included studies. The study populations included infants, preschool children, school-age children, and prepubertal children; the interventions included different ICS formulations such as beclomethasone dipropionate, fluticasone propionate, and budesonide; and the comparators included placebo, no ICS treatment, montelukast, and other nonsteroidal controller therapies (5, 8–10, 18, 20–27).

In this review, reduced linear growth velocity appeared more evident within school-age children than within infants. This may reflect more stable growth patterns and more reliable measurement in school-age populations, whereas growth in infancy is influenced by multiple factors including nutrition, infection, and developmental variability. However, because the formal test for subgroup differences was not statistically significant, age should not be interpreted as a confirmed effect modifier.

Similarly, no statistically significant subgroup difference was observed between fluticasone and beclomethasone. This absence of a significant difference should not be interpreted as evidence of equivalent effects among ICS formulations, because the subgroup analyses were limited by small study numbers, variation in dose and delivery device, and incomplete information on adherence and cumulative exposure. Therefore, the subgroup analyses should be viewed as exploratory. They partially examined potential sources of heterogeneity but did not fully account for the residual variability observed in the primary analysis.

### 4.3 Interpretation by comparator

Asthma severity is an important potential confounder when interpreting growth outcomes. Children with more severe or poorly controlled asthma are more likely to receive higher-dose or longer-duration ICS therapy, but the underlying disease itself may also affect growth through systemic inflammation, reduced physical activity, sleep disturbance, impaired nutrition, and cumulative exposure to oral corticosteroids (29). Therefore, observed differences in growth cannot always be attributed to ICS alone, particularly when asthma severity, disease control, adherence, and oral corticosteroid exposure are incompletely reported or controlled.

Comparator type may also influence the observed effect size. Children receiving placebo or no regular controller therapy may have poorer asthma control, whereas children receiving active non-ICS therapies such as montelukast may differ in both treatment exposure and disease control. Therefore, comparator-related findings should be interpreted cautiously and considered hypothesis-generating rather than definitive.

### 4.4 Effect on adult height

The available evidence on adult height suggests that ICS therapy during childhood may be associated with an approximately 1.2-cm reduction in adult height. Although this estimate reached statistical significance, its interpretation requires caution. On the one hand, it supports the possibility that early treatment-related slowing of height gain may not always be followed by complete catch-up growth. On the other hand, the absolute magnitude of the observed difference was small and was derived predominantly from a single long-term follow-up study, which limits both robustness and generalizability.

The clinical significance of these findings should be interpreted carefully. Although some analyses suggested small reductions in linear growth velocity or adult height, the absolute magnitude of the observed effects was limited, and the primary meta-analysis for linear growth velocity did not reach statistical significance. For most children with persistent asthma, the established benefits of ICS in improving symptom control, preventing exacerbations, and reducing asthma-related morbidity are likely to outweigh a small and uncertain potential effect on growth. Therefore, these findings support individualized dose optimization, correct inhalation technique, and longitudinal growth monitoring rather than avoidance of clinically indicated ICS therapy.

These findings suggest that ICS therapy should continue to be individualized according to asthma control and safety considerations (30). Clinicians should use the lowest effective dose, reinforce correct inhalation technique, review adherence regularly, and monitor growth longitudinally in children requiring prolonged ICS treatment.

### 4.5 Strengths and limitations

This study has several strengths, including the focus on a clinically important question, inclusion of randomized controlled trials, and integration of both short-term and long-term growth outcomes. The use of GRADE further enhances interpretability.

However, several limitations should be considered. First, PROSPERO registration was completed after the database and trial registry searches had been conducted. Registration was completed before final study selection, data extraction, risk-of-bias assessment, and quantitative synthesis. Second, the number of included studies was limited, and only a subset contributed to meta-analysis. Third, substantial heterogeneity existed in age, ICS exposure, comparators, follow-up duration, and outcome definitions. Fourth, incomplete reporting in some studies may have affected the robustness of pooled estimates and also limited the certainty of some RoB 2 domain judgments, particularly for the randomization process and selection of the reported result. Fifth, because the main synthesis was restricted to randomized trials and long-term follow-up reports derived from randomized trial cohorts, independent prospective cohort studies were excluded. Although this approach reduced confounding by indication and improved internal validity, it may also have limited the amount of long-term safety evidence available, particularly for adult or near-adult height. Sixth, adult height outcomes were derived largely from a single long-term study (9). Seventh, limited study numbers precluded meta-regression analyses of dose-response relationships and other modifiers. Finally, publication bias could not be formally assessed due to the small number of studies.

### 4.6 Future research directions

Further well-designed studies with larger sample sizes and longer follow-up are needed to clarify the effects of different ICS formulations, dosing strategies, and age at treatment initiation on both linear growth velocity and adult height in children (31). Future research should also consider pubertal stage, cumulative exposure, inhalation technique, adherence, and disease control to better distinguish treatment effects from disease-related influences. Standardized reporting of growth outcomes, including linear growth velocity, height SDS/Z-score, and adult height, would further improve the quality and comparability of evidence.

### 4.7 Concluding discussion

Taken together, the primary meta-analysis did not demonstrate a statistically significant reduction in linear growth velocity among children receiving long-term maintenance ICS therapy. The statistically significant result observed after exclusion of studies at high risk of bias suggests a possible small reduction in linear growth velocity, but this should be regarded as supportive rather than definitive evidence. Evidence for adult height was limited and derived predominantly from a single long-term follow-up study, and therefore remains uncertain.

Overall, these findings suggest that long-term ICS therapy may have a small growth-related effect in some children, but the certainty of evidence is limited by moderate heterogeneity, small numbers of quantitatively pooled studies, variability in age, ICS formulation, dose, comparator, and follow-up duration, and limited long-term outcome data. Accordingly, the clinical implication is not to withhold necessary ICS treatment, but to optimize individualized asthma management, use the lowest effective dose, reinforce correct inhalation technique, and monitor growth longitudinally.
