# Supplementary material for: Effects of long-term inhaled corticosteroids on growth velocity and adult height in children with asthma: a systematic review and meta-analysis
Source: Front Med (Lausanne). 2026 Sep 14;13:1937498. doi: 10.3389/fmed.2026.1937498 (PMC13617044; doi:10.3389/fmed.2026.1937498)
Supplement: Supplementary file 1 [file Data_Sheet_1.docx]

Appendices

Appendix S1: Search Strategies

Systematic Review Title: The Impact of Long-Term Inhaled Corticosteroid Maintenance Therapy on Linear Growth in Children with Asthma: A Systematic Review and Meta-Analysis

Date of final search: 26 March 2026. All electronic database and trial registry searches were completed on this date, one day before PROSPERO registration on 27 March 2026.

The following databases were searched: PubMed, Embase, Cochrane CENTRAL, CNKI, WanFang, Scopus, and SinoMed. Trial registries including ClinicalTrials.gov and WHO ICTRP were additionally searched to identify ongoing or unpublished studies.

| Database | Platform | Search String | Results |
| --- | --- | --- | --- |
| PubMed | [PubMed.gov](https://pubmed.gov/) | (("Asthma"[Mesh] OR asthma[tiab] OR wheez[tiab]) AND ("Child"[Mesh] OR "Adolescent"[Mesh] OR child[tiab] OR pediatric[tiab] OR paediatric[tiab] OR infant[tiab] OR adolescent[tiab]) AND (("Growth"[Mesh] OR "Body Height"[Mesh] OR growth[tiab] OR height[tiab] OR stature[tiab] OR "linear growth"[tiab] OR "growth velocity"[tiab] OR "height z score"[tiab] OR "height SDS"[tiab])) AND (("Adrenal Cortex Hormones"[Mesh:NoExp] OR "Anti-Asthmatic Agents"[Mesh] OR "inhaled corticosteroid*"[tiab] OR ICS[tiab] OR beclomethasone[tiab] OR budesonide[tiab] OR fluticasone[tiab] OR mometasone[tiab] OR ciclesonide[tiab])) AND (("Randomized Controlled Trial"[pt] OR "Controlled Clinical Trial"[pt] OR "Cohort Studies"[Mesh] OR randomized[tiab] OR randomised[tiab] OR trial[tiab] OR cohort[tiab] OR "longitudinal"[tiab] OR prospective[tiab]))) | 174 |
| Embase | [Embase.com](https://embase.com/) | (('asthma'/exp OR asthma:ti,ab OR wheez:ti,ab) AND ('child'/exp OR 'adolescent'/exp OR child:ti,ab OR pediatric:ti,ab OR paediatric:ti,ab OR adolescent:ti,ab OR infant:ti,ab) AND ('growth'/exp OR 'body height'/exp OR growth:ti,ab OR height:ti,ab OR stature:ti,ab OR 'linear growth':ti,ab OR 'growth velocity':ti,ab) AND ('inhaled corticosteroid'/exp OR 'anti asthmatic agent'/exp OR 'inhaled corticosteroid*':ti,ab OR ICS:ti,ab OR beclomethasone:ti,ab OR budesonide:ti,ab OR fluticasone:ti,ab) AND ('randomized controlled trial'/exp OR 'cohort analysis'/exp OR randomized:ti,ab OR randomised:ti,ab OR 'clinical trial':ti,ab OR cohort:ti,ab OR prospective:ti,ab)) | 294 |
| Cochrane CENTRAL | Cochrane Library | ((MeSH descriptor: [Asthma] explode all trees OR (asthma or wheez):ti,ab,kw) AND (MeSH descriptor: [Child] explode all trees OR (child or pediatric or paediatric or adolescent* or infant):ti,ab,kw) AND (MeSH descriptor: [Growth] OR [Body Height] explode all trees OR (growth or height or stature or "linear growth" or "growth velocity"):ti,ab,kw) AND ((inhaled NEXT corticosteroid or ICS or beclomethasone or budesonide or fluticasone or mometasone or ciclesonide):ti,ab,kw)) | 281 |
| CNKI | [CNKI.net](https://cnki.net/) | SU=('哮喘' + '支气管哮喘') AND SU=('儿童' + '小儿' + '婴幼儿' + '青少年') AND SU=('生长' + '身高' + '发育' + '生长速率') AND SU=('吸入激素' + '糖皮质激素' + '布地奈德' + '氟替卡松' + 'ICS') AND (SU=('随机对照试验' + '队列研究' + '前瞻性研究') OR FT=('随机' + '对照' + '队列')) | 93 |
| Wanfang | [Wanfangdata.com.cn](https://wanfangdata.com.cn/) | 主题/题名/关键词: (哮喘 OR 支气管哮喘) AND (儿童 OR 小儿 OR 婴幼儿 OR 青少年) AND (生长 OR 身高 OR 发育 OR 生长速率) AND (吸入激素 OR 吸入性糖皮质激素 OR 布地奈德 OR 氟替卡松 OR ICS) AND 全部字段: (随机对照试验 OR 随机分配 OR 队列研究 OR 前瞻性研究) | 89 |
| SinoMed | [Sinomed.ac.cn](https://sinomed.ac.cn/) | (哮喘[不加权:扩展] OR (哮喘 OR 支气管哮喘 OR 喘息)) AND (儿童[不加权:扩展] OR (儿童 OR 小儿 OR 婴幼儿 OR 青少年)) AND (生长[不加权:扩展] OR 身高[不加权:扩展] OR (生长 OR 身高 OR 发育 OR 生长速度)) AND (糖皮质激素类/投药和剂量[不加权:扩展] OR (吸入激素 OR 吸入性糖皮质激素 OR 布地奈德 OR 氟替卡松 OR ICS)) | 122 |
| [ClinicalTrials.gov](https://clinicaltrials.gov/) | [clinicaltrials.gov](https://clinicaltrials.gov/) | Condition: Asthma; Other terms: child OR pediatric; Intervention/treatment: inhaled corticosteroid OR budesonide OR fluticasone | 0 |
| WHO ICTRP | [trialsearch.who.int](https://trialsearch.who.int/) | asthma AND child AND inhaled corticosteroid | 0 |
| Scopus | Scopus.com | TITLE-ABS-KEY ((asthma OR asthmatic* OR wheez*) AND (child* OR pediatric* OR paediatric* OR adolescen* OR infant* OR preschool*) AND (growth OR height OR stature OR "linear growth" OR "growth velocity" OR "height z score" OR "height SDS" OR "height standard deviation score") AND ((inhal* W/3 corticosteroid*) OR (inhal* W/3 glucocorticoid*) OR ICS OR beclomethasone OR budesonide OR fluticasone OR mometasone OR ciclesonide) AND (random* OR trial* OR "controlled clinical trial" OR cohort OR longitudinal OR prospective)) | 666 |
| Total |  |  | 1,719 |

Appendix S2. Studies included in qualitative synthesis only and reasons for exclusion from quantitative synthesis

| Study | Comparison / growth-related outcome reported in Table 1 | Reason for retention in qualitative synthesis only |
| --- | --- | --- |
| Visser et al., 2004 [27] | High-to-low fluticasone propionate vs fixed-dose fluticasone propionate; linear growth velocity | Compared two fluticasone propionate dosing strategies rather than ICS versus placebo, no regular controller therapy, or a non-ICS controller. The comparator structure was therefore not compatible with the prespecified pooled comparisons. |
| Visser et al., 2001 [26] | Step-down fluticasone propionate vs fixed-dose fluticasone propionate; height | Compared two fluticasone propionate dosing schedules. The reported height outcome was not sufficiently comparable with the linear growth velocity outcome used in the primary quantitative synthesis. |
| CAMP, 2000 ^[8]^ | Budesonide 400 micrograms/day vs placebo; height | Reported short- to medium-term height outcomes, but did not provide extractable linear growth velocity data compatible with the primary pooled analysis. Long-term adult-height evidence from the same trial cohort was represented by Kelly et al. [9], avoiding double counting of the same cohort for the same outcome domain. |
| Sorkness et al., 2007 ^[25]^ | Fluticasone propionate plus/minus LABA vs montelukast; growth-related outcome | The intervention involved a combined controller regimen, and the reported growth-related data were not sufficiently compatible with the prespecified pooled comparisons. |
| Martinez et al., 2011 ^[24]^ | Daily or rescue beclomethasone dipropionate vs placebo; height | Reported height-related outcomes, but the intervention structure and reporting format were not sufficiently compatible with the primary linear growth velocity analysis or the adult-height analysis. |
| Guilbert et al., 2006 ^[18]^ | Fluticasone propionate 88 micrograms twice daily vs placebo; height | Reported height-related outcomes in preschool children, but the available data were not in a format directly compatible with the prespecified quantitative syntheses of linear growth velocity or adult/near-adult height. |
| Chen et al., 2001 ^[20]^ | Beclomethasone dipropionate 200/400 micrograms/day vs placebo; height SDS and BMD | Reported height SDS and bone mineral density rather than extractable linear growth velocity or adult/near-adult height data with sufficient summary statistics for quantitative pooling. |

Abbreviations: BDP, beclomethasone dipropionate; BMD, bone mineral density; FP, fluticasone propionate; ICS, inhaled corticosteroids; LABA, long-acting beta2-agonist; SDS, standard deviation score.

Appendix S3. Leave-one-out sensitivity analysis for the primary outcome of linear growth velocity

| Study report | Pooled MD (cm/year) | 95% CI | P value | I2 (%) |
| --- | --- | --- | --- | --- |
| Piao, 2011 ^[21]^ | -0.50 | -0.96 to -0.04 | 0.034 | 58.0 |
| Allen et al., 1998 ^[10]^ | -0.34 | -0.93 to 0.25 | 0.260 | 69.8 |
| Allen et al., 1998 ^[10]^ | -0.41 | -0.95 to 0.13 | 0.135 | 65.3 |
| Doull et al., 1995 ^[5]^ | -0.20 | -0.53 to 0.12 | 0.220 | 0.0 |
| Wang et al., 1999 ^[22]^ | -0.38 | -0.87 to 0.12 | 0.136 | 70.9 |
| Wang et al., 1999 ^[22]^ | -0.44 | -0.91 to 0.03 | 0.064 | 67.2 |

Abbreviations: BDP, beclomethasone dipropionate; CI, confidence interval; FP, fluticasone propionate; ICS, inhaled corticosteroids; MD, mean difference.
